# An Emerging Health Crisis in Turkey and Syria after the Earthquake Disaster on 6 February 2023: Risk Factors, Prevention and Management of Infectious Diseases

**DOI:** 10.3390/healthcare11071022

**Published:** 2023-04-03

**Authors:** Maria Mavrouli, Spyridon Mavroulis, Efthymios Lekkas, Athanassios Tsakris

**Affiliations:** 1 Department of Microbiology, Medical School, National and Kapodistrian University of Athens, 11527 Athens, Greece; 2 Department of Dynamic Tectonic Applied Geology, Faculty of Geology and Geoenvironment, School of Sciences, National and Kapodistrian University of Athens, 15784 Athens, Greece; 3 Earthquake Planning and Protection Organization, 15451 Athens, Greece

**Keywords:** disaster, crisis, earthquakes, public health, health emergencies, infectious diseases, surveillance, disaster preparedness

## Abstract

On 6 February 2023, Turkey and Syria were hit by two major earthquakes that caused extremely heavy structural damage to buildings and infrastructure in one of the most densely populated areas of Anatolia. The authors visited the devastated area shortly after the earthquakes in the frame of search and rescue and scientific missions in order to check whether the newly formed conditions have the potential to further affect public health. Based on the collected disaster-related field data, it is revealed that risk factors associated with and favoring emergence of infectious diseases are present in the affected residential areas from the first hours of the emergency state. The coexistence and synergy of many collapsed health facilities, cold winter conditions, destruction of lifeline infrastructures, overcrowding in emergency shelters, poor sanitation and adverse socio-economic conditions along with evolving crises and disasters (conflicts, pandemic and epidemics) may further aggravate the already fragile public health situation and cause considerable delays in the recovery process. Efficient disease surveillance at local and regional levels is a crucial requirement for early warning and protection against emerging infectious diseases in the earthquake-affected areas among other proposed measures for prevention and management of infectious diseases.

## 1. Introduction

**Figure 1:**
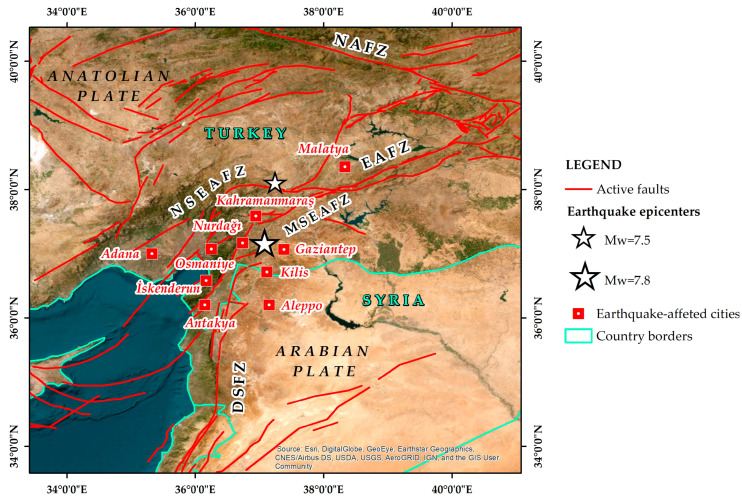
Map illustrating the area where the 6 February 2023 earthquake disaster took place. The Mw = 7.8 earthquake epicenter was determined west of Gaziantep and the Mw = 7.5 earthquake epicenter northeast of Kahramanmaraş. Both earthquakes were generated within the East Anatolian Fault Zone (EAFZ), which constitutes a major active left-lateral strike-slip fault zone between the Anatolian Plate and the northward-moving Arabian Plate (NSEAFZ: North strand of EAFZ; MSEAFZ: Main strand of EAFZ; DSFZ: Dead Sea Fault Zone; NAFZ: North Anatolian Fault Zone) Most affected cities are also presented. Faults (red lines) are based on Styron and Pagani [3].

On 6 February 2023, Turkey and Syria were hit by a devastating Mw = 7.8 earthquake generated by the rupture of the southeastern part of the East Anatolian Fault Zone [1] (Figure 1). Based on several seismological institutes and observatories, its epicenter was located westwards of Gaziantep city and its focal depth reveals a shallow event [1]. It triggered the generation of a new Mw = 7.5 earthquake within the same fault zone, at a distance of 100 km further north (Figure 1).

These earthquakes caused extensive primary and secondary effects on the environment including surface ruptures, ground cracks, liquefaction phenomena, tsunami and hydrological anomalies causing extensive impact on networks and infrastructure [2], phenomena that gave local and foreign scientists who visited the area the opportunity to gain a comprehensive picture of the seismotectonic regime and the intensity of the accompanied phenomena.

However, the most important impact of the generated earthquakes was the extremely heavy structural damage to elements of the built environment and especially to buildings and infrastructure in one of the most densely populated areas of Anatolia [2]. Hundreds of thousands of buildings collapsed and thousands of residents staying in their apartments in high-rise blocks of flats lost their lives, while for those who remained trapped but alive in the rubble a battle against time and adverse conditions began until they were rescued. As of this writing (13 February 2023), the Disaster and Emergency Management Presidency (AFAD) has reported 31,643 human casualties, while 158,165 people have been evacuated from the 10 earthquake-affected provinces [4], while in Syria, over 5000 human casualties have been reported with these numbers expected to increase as the search and rescue operations reach completion. According to a report of the World Health Organization (WHO) [5], 15 million people are affected in Turkey and 10.87 million people affected in Syria. Almost 26 million people are exposed of whom 5.08 million could be characterized as vulnerable comprising over 345,000 elderly and over 1.4 million children [5].

However, a new threat is emerging for the local population remaining in the affected area and for those involved in managing the adverse conditions including rescue teams, health workers, volunteers and crews working for hazard mitigation in the aftershock period. This threat is related to public health and its burden from sporadic cases, outbreaks and epidemics of infectious diseases.

The aim of this study is to highlight all risk factors that favor the emergence of infectious diseases in the earthquake-affected area of southeastern Turkey and northern Syria. This will be achieved not only by taking into account the significant results of existing relevant studies, but mainly by presenting field data obtained from post-event field surveys, conducted by the authors in the earthquake-affected area shortly after the earthquake, as well as from their participation at different stages of the disaster management cycle, especially during the emergency response. Moreover, this study aims to provide an important tool in the hands of scientists, health professionals and civil protection and disaster management personnel to effectively prevent, control and manage potential infectious disease outbreaks in the post-disaster period. These measures represent good practices and lessons learned from a similar succession of past geophysical and biological hazards (earthquakes and infectious diseases, respectively) either in these countries or worldwide. These measures fit on a multi-hazard approach, which has become a common strategy in recent years on a global scale due to the evolving COVID-19 pandemic.

## 2. Methodology

The methodology applied for highlighting the risk factors favoring the emergence of a health crisis in the areas affected by the 6 February 2023 earthquake events comprises initially the collection of disaster-related data during post-event surveys conducted by the authors (E.L. and S.M.) in the field. The third author of the paper, E.L., visited the earthquake-affected area shortly after the destructive events as the President of the Earthquake Planning and Protection Organization of Greece (EPPO, OASP in Greek) and a member of the Greek search and rescue (SAR) mission, which responded directly to Turkey’s request for immediate assistance [6]. The Greek SAR mission comprised the 1st and 2nd Disaster Management Special Units (EMAK in Greek) of the Hellenic Fire Service, officer-engineers of the Hellenic Fire Service, doctors and rescuers of the National Center of Emergency Assistance of Greece (EKAB in Greek) and the President of the EPPO [6]. They operated in Hatay province and returned to Greece on 13 February having rescued five people alive and recovered five unconscious people [6].

The second author, S.M., accompanied E.L. in the disaster field as a member of the scientific mission of the Department of Geology and Geoenvironment of the National and Kapodistrian University of Athens (Greece) and stayed in the area collecting disaster-related data for five consecutive days after the occurrence of the devastating earthquakes (7–11 February).

The researchers collected disaster-related data that had to do with the impact on buildings, including damage to health facilities, the impact on the roads, drinking-water and electricity supply networks, as well as the main response actions during the emergency phase, comprising the distribution of sufficient basic necessities to the affected people and the selection and adequacy of shelters for temporary housing of the homeless.

The methodology was applied to many segments of the earthquake-affected area including devastated urban areas with a population ranging from hundreds of thousands to millions of people, thousands of fatalities and millions of affected people. The largest affected urban centers visited by the researchers from north to south are the cities of Kahramanmaraş, Nurdağı, Gaziantep, Osmaniye, Adana, Iskenderun and Antakya (Figure 1).

The collected data were then evaluated by all authors. The risk factors prevailing in the region that may favor the emergence of infectious diseases were first recognized and then the most effective measures for their prevention and management were selected and proposed as a significant tool for health officials, scientists and operators involved in the emergency response and recovery phase.

In the context of this approach, a review comprising only examples of infectious disease outbreaks following past earthquakes in different regions of Turkey was conducted. These regions present many similarities in the seismotectonic regime, population characteristics and demographic properties, and earthquake-triggered effects on the natural and built environment, but differences in the intensity and extent of the triggered effects. Although smaller than those triggered by the February 2023 earthquakes, they had the potential to cause infectious disease outbreaks.

## 3. Results

### 3.1. Infectious Diseases Emerged after Previous Earthquakes in Turkey

Similar adverse conditions following a devastating earthquake have occurred before in Turkey. Based on EM-DAT, the 17 August 1999, Mw = 7.6 Izmit earthquake generated in northwestern Turkey caused 17,127 casualties, 43,953 injured and 1,358,953 total affected people, while the 23 October 2011, Mw = 7.1 Van earthquake in eastern Turkey caused 604 casualties, 4152 injured and 32,938 total affected people [7]. After these earthquakes, the synergy of the above risk factors led to the occurrence of infectious diseases including gastrointestinal infections (water- and food-borne diseases), and wound and skin infections [8].

Immediately after the 1999 Izmit (Turkey) earthquake, an infectious disease surveillance system was established in Kocaeli province, which was devastated by the earthquake. Waterborne microorganisms identified prior to the earthquake, such as *Shigella* species, *Salmonella* species, and *Giardia* intestinalis, could be the possible causative agents of diarrhea outbreaks triggered by the hot summer weather, destruction of infrastructure, and barriers to obtaining safe drinking water. Diarrheal diseases increased gradually after 20 August 1999, and then reverted to normal level on 15 September 1999. *Shigella* species were the most prevalent isolates among the listed causes [9].

Following the 1999 Izmit earthquake, inconsistencies were observed in the drinking water supply and the maintenance of adequate sanitation and hygiene conditions in the emergency shelters, which led to Hepatitis A and E incidence increase in children living in camps [10]. After the 1999 Düzce earthquake, generated a few months after the Izmit event, the necessary preventive measures, including setting up of emergency shelters, provision of clean drinking water and food and distribution of financial aid, were quickly implemented. The immediate response and implementation of measures led to a reduced prevalence of Hepatitis A and E in children in camps of Düzce district compared to that observed after the Izmit earthquake. It is worth mentioning that even four years after the earthquake in the city of Düzce, Hepatitis A was still prevalent in pediatric age groups, whereas Hepatitis E occurred relatively rarely [11].

Even prolonged staying in temporary settlements after devastating earthquakes could contribute to the emergence and persistence of infectious diseases. By comparing two groups of children living and studying in different socioeconomic conditions due to the Düzce earthquake, it was found that the incidence rate of giardiasis and enterobiasis was significantly higher in children who were still living in and attending temporary settlements and schools even years after the earthquake compared to children with a higher socioeconomic background, living and studying in normal conditions [12].

Regarding skin infections associated with the 1999 Izmit earthquake, it was demonstrated that damaged infrastructure and unhealthy living conditions contributed to the occurrence of infections/infestations and dermatoses, while psycho-emotional factors related to the earthquake favored the development of pruritus, erythemato-squamousdiseases, eczemas, and neurocutaneous dermatoses [13].

Traumatic injuries and crush syndrome that occur after an earthquake increase the predisposition to infectious complications and may cause septic shock and eventually multiple organ failure [14]. Most of the wound infections recorded following the 1999 Izmit and the 2011 Van earthquakes were hospital-acquired and were mainly caused by resistant Gram-negative aerobic bacteria. *Acinetobacter baumannii* was the most common causative agent of wound infections among victims of both earthquakes [14,15,16].

### 3.2. Risk Factors for Emergence of Infectious Diseases in the 2023 Earthquake-Affected Area of East Anatolia

From the very first hours after the occurrence of the devastating earthquakes, the affected areas of Turkey and Syria concentrated many risk factors for the emergence of infectious diseases.

**Figure 2:**
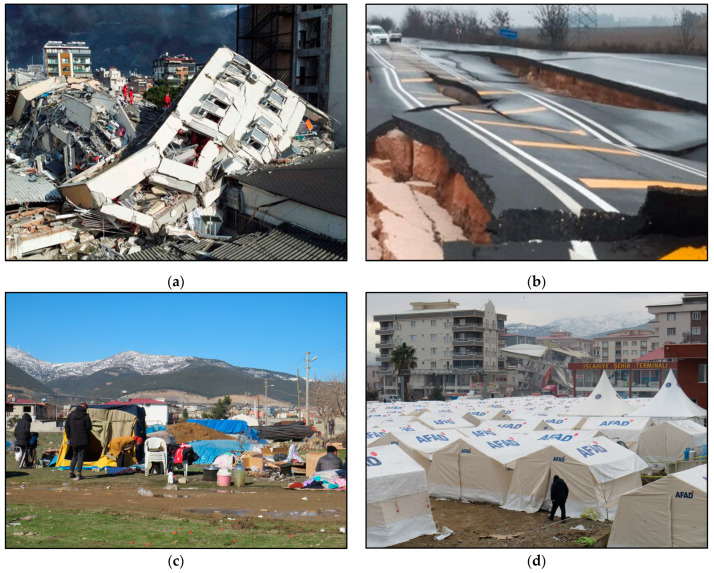
(**a**) The collapsed state hospital in İskenderun. (**b**) Extensive cracking and total destruction of the road due to the earthquake ground shaking resulting in permanent traffic disruption. (**c**) Emergency shelter set up by AFAD in Islahiye city. (**d**) A large part of the affected population in Turkey and Syria face adverse conditions formed by the devastating 6 February earthquakes. Lack of fresh water and emergency supplies are favorable for the emergence of infectious diseases. Photos taken during post-event field survey conducted in the field by E. Lekkas and S. Mavroulis from 7 to 11 February 2023.

Many health facilities including state hospitals suffered severe structural damage with some of them collapsing. According to data presented by the WHO [5], 15 hospitals in Turkey have suffered partial or heavy damage and 48 health facilities were affected in northwest Syria (Figure 2a).

These collapses resulted in fatal injuries, not only to the patients being treated, but also to the doctors and the nursing staff working in these facilities at the time of the earthquakes. These human losses may lead to a reduction in the number of medical personnel who could have directly contributed to the rehabilitation and relief of the affected people, but also increase the time required for immediate medical care. Furthermore, health facilities on the verge of collapse have all been evacuated. People injured by debris had to be transported to hospitals in neighboring provinces and towns, which are tens and hundreds of kilometers away from the earthquake-affected areas. The evacuation time becomes even longer if it is taken into account that sections of the road network near the residential areas were either destroyed by the intense ground-shaking (Figure 2b) or particularly burdened due to increased traffic of emergency vehicles including ambulances, fire-fighting trucks, police patrol vehicles, etc., heavy vehicles such as crane trucks, excavators, loaders, etc., as well as many private vehicles of residents and visitors.

The already difficult situation created by the earthquakes with tens of thousands of dead and injured was further aggravated by the difficult weather conditions in the region. The severe winter in the area is characterized by low temperatures during the day and even lower at night (between −10 and 10 °C) and snow in the semi-mountainous and mountainous parts. These conditions make it difficult for those trapped in the rubble to survive, as they are exposed to low temperatures among other things, but also for the homeless, many of whom chose to stay near their completely destroyed homes for fear of imminent looting. The only solution for the hundreds of thousands of homeless people is the emergency shelters set up in the days after the earthquakes in open spaces, such as open football stadiums and empty fields, far away from destroyed buildings.

As regards the affected infrastructure, the water supply, electricity, telephone and product distribution networks are completely destroyed in most cities. The rupture of water supply and sewerage pipes led not only to a permanent interruption in the supply of drinking water to the affected people (Figure 2c), but also to contamination of the available water sources, leading to an increase in the risk of gastrointestinal infectious diseases (water- and food-borne diseases). The disruption of commercial activities results in insufficient food provision and shortages of long-lasting food supplies as well as shortages of personal hygiene items, which may lead to malnutrition of the affected population and unsanitary conditions (poor hand hygiene), respectively.

The 6 February 2023 earthquakes created tens of thousands of temporarily homeless people, whose homes have mostly collapsed or suffered severe structural damage, preventing them from returning to their homes. A large proportion of the temporarily homeless are accommodated in emergency shelters organized by AFAD [17] (Figure 2d). The large number of people in emergency shelters and evacuation camps can create the ideal conditions for the outbreak of infectious diseases in the post-earthquake period, as evidenced by lessons learned from recent destructive earthquakes [8]. Influenza can have a significant public health impact in Turkey and Syria since the earthquakes occurred during the transmission period. Additionally, SARS-CoV-2 still circulating in the earthquake-affected areas can increase the number of COVID-19 cases [18]. Overcrowding into small spaces without maintaining proper physical distance, insufficient basic items for the homeless and evacuees, inadequate heating, ventilation, and air conditioning systems, unsafe drinking water, malnutrition brought on by inadequate food provision and a lack of long-lasting food supplies, and poor personal hygiene are among the potential risk factors for developing respiratory infectious diseases in evacuation shelters [8].

Examples of sporadic cases, outbreaks, and epidemics of infectious diseases are frequently drawn from Japan and the emergency shelters that were constructed to house those in need of shelter and food after the 2011 earthquake and tsunami that devastated a large part of the country’s eastern coast [19,20]. Emergency shelters were so crowded that evacuees were compelled to lie on their backs on the floor, unable to roll over while sleeping. Some of them even hesitated to cough to avoid upsetting those nearby, while others neglected their oral hygiene. People, especially the elderly and infants, developed pneumonia as a result of the aforementioned conditions and the synergistic effects of frequent aspiration, malnutrition, and low temperatures [19,21].

Infectious disease transmission in populations is a direct result of the connection and interaction among the three elements of the Epidemiologic Triangle: a pathogenic agent, a susceptible host, and an environment where pathogen and host encounter each other [22]. Due to changing patterns of contact among humans, pathogens, and rodents during the post-earthquake period, rodent-borne diseases may potentially become more prevalent. The affected people can become infected by directly coming into contact with infected animal hosts such as rodents, domestic pets and livestock, as well as by being exposed to water, food, or soil contaminated by the urine of infected animals. Garbage accumulation, poor waste management practices and rat infestations all contribute to leptospirosis occurrence among evacuees. It is critical to recognize and identify local rodent and vector species, environmental factors, and breeding habitats that affect local disease transmission in order to implement response activities, control measures, and enhance preparedness efforts for emerging mosquito- and rodent-borne diseases.

Furthermore, the situation in northwestern Syria is particularly severe due to nearly 12 years of war and the additional consequences of a volatile economic situation, the COVID-19 pandemic and, more recently, a cholera epidemic, which has been associated with the consumption of contaminated water from the Euphrates River in northeastern Syria. These variables make it even more challenging to handle the current emergency in Syria, as the health care system was already fragile and reliant on humanitarian aid [23].

Disruption of surveillance and health care programs, such as immunization or vector control programs, can create ideal conditions conducive to transmitting vaccine-preventable or vector-borne diseases. According to Sahinoz et al. [24], between 1960 and 2019 1,050,567 measles cases were recorded in Turkey. Unvaccinated children, young children who have not reached the vaccination age, persons who refuse vaccinations, and imported cases make up the majority of cases observed in Turkey in recent years [24,25]. Measles epidemics recorded in 2017 and 2018 were the worst since Syria declared the disease eradicated in 1999. Intensely conflicted and displaced communities were most affected by these outbreaks. The measles spread in northern Syria is a sign of low immunization rates and poor access to healthcare, and demonstrates how susceptible the Syrian people are to infectious diseases and diseases that may be prevented by vaccination [26]. In addition, the interruption of ongoing treatments, such as those necessary to treat tuberculosis, and the use of over-the-counter medicines due to limited access to healthcare services can increase disease severity and treatment burdens and contribute to the subsequent high incidence of disease transmission.

## 4. Discussion

One of the most effective measures for prevention of earthquake-induced infectious disease outbreaks is the establishment of a proper disease surveillance system. Its main purpose is to rapidly identify the post-disaster sporadic cases of infectious diseases by initially collecting and analyzing relevant information and then to improve disease trends monitoring and validity of early warnings and thus to more fully assess the public health burden of infectious diseases [8]. Infectious disease surveillance is a crucial epidemiological tool for monitoring a population’s health that interprets collected health-related data in a continuous and systematic manner [27,28,29]. The three objectives of infectious disease surveillance are as follows:

- The description of the current disease burden and epidemiology, which is critical as it demonstrates and supports the need for public health measures and interventions (e.g., vaccination);
- The monitoring and analysis of disease trends associated not only with numbers of cases but also with causative pathogenic agents;
- The detection of outbreaks and new pathogens.

Emerging diseases caused by pathogens appearing in a population for the first time, and re-emerging diseases, caused by known pathogens that expand geographically or are reintroduced into the population, constitute a major threat to public health [28]. Ongoing surveillance for epidemic-prone diseases and timely dissemination of results can aid in early detection of an outbreak, allowing for a more rapid response and, thus, mitigation of outbreaks with the potential to become public health emergencies. Depending on the epidemiology and clinical presentation of the disease, as well as the goals of surveillance, different approaches to infectious disease surveillance may be used [28].

The occurrence of infectious diseases while staying in emergency shelters can be avoided by providing ample quantities of bottled water, canned and dry food and adequate ventilation (heating and air conditioning), distributing personal protective equipment (face mask, disposable gloves, disinfectants), and making available sufficient quantities of appropriate medical supplies, pharmaceutical products and effective vaccines [8]. Hygiene awareness-raising and sanitation promotion methods should be applied for evacuees and staff. Furthermore, materials comprising leaflets and posters on enhanced prevention of infectious diseases should be displayed in various sites in the emergency shelters and in the affected community [8]. Individual toilets should be used instead of communal ones and camp areas should have trench latrines to avoid defecation in the open. Hand hygiene is essential, ensured by hand-washing with soap and water.

More emergency shelters of the same or different type, such as campervans, container structures, hotel rooms, residences of relatives and close friends, in addition to open fields and football stadiums, should be utilized to prevent overcrowding in emergency shelters [30,31]. When several countries were devastated by earthquakes during the COVID-19 pandemic, this strategy was successfully used to keep physical distance between evacuees in an effort to limit the spread of SARS-CoV-2 in the communities that had been affected by the earthquake [30,31]. Additionally, the majority of evacuation shelters should be sufficiently equipped with heating sources as it is well known that hypothermia can increase the risk of respiratory infections, including pneumonia [32]. As soon as evacuees gather in camps, vaccines such as MMR (measles, mumps and rubella), flu, *Haemophilus influenzae* type b, pneumococcal and polio vaccines should be administered to prevent the occurrence and rapid spread of measles, influenza, meningitis and poliomyelitis. Furthermore, a proper disease surveillance program should be implemented along with a long-term strengthening and reinforcement of the public sector healthcare system. The World Health Organization (WHO) and the United Nations Children’s Fund (UNICEF) along with Syrian health authorities began a cholera vaccination campaign in earthquake-affected areas of Northwest Syria [33].

One of the most effective measures suggested to prevent the occurrence of waterborne diseases during the emergency in the earthquake-affected area of East Anatolia is the examination of water supply and sewerage systems to detect non-structural and structural failures that are likely to affect public health [8]. This is a practice that has been applied in all developed countries for decades. If non-structural and structural defects are detected in these systems, chlorination or even an initial supply from a different source is applied, followed by disinfection [34,35]. In addition, the safety of drinking-water and sanitation systems should be also ensured in temporary emergency shelters or permanent relief camps. Where existing safety and hygiene measures are insufficient, educational programs should be implemented to enhance public knowledge of sanitation and hygiene issues [8].

Public health officials should prioritize surveillance to reduce the risk of rodent-borne infections in all the earthquake-affected regions of southeastern Turkey. The immediate detection and identification of local rodent species, environmental variables and breeding habitats that affect local disease transmission is essential for the implementation of control measures and the advancement of preparedness initiatives aimed at emerging rodent-borne diseases [36]. The earthquake-affected population should also avoid places such as uncontrolled waste disposal sites and stagnant water areas where they may come into contact with infectious material, such as contaminated surface water or soil and contaminated animal urine, and be exposed to various pathogenic microorganisms [37].

Knowledge of modes of disease transmission and how pathogenic microorganisms multiply and spread can enhance the implementation of measures and efforts to control and prevent infectious diseases [38]. The general public and emergency management personnel should be informed on how infectious diseases are transmitted, how to identify their symptoms and how to seek immediate medical attention and guidance in case of illness. Early detection of signs and symptoms and application of appropriate treatment are crucial to reducing morbidity and mortality since incorrect or delayed diagnosis has serious clinical implications.

It is important to note that disasters brought on by natural hazards do not introduce new diseases to the affected areas. The transmission of an infectious agent can actually occur if it is endemic to the affected area or if it is brought there through search-and-rescue operations [39,40]. When environmental conditions become favorable, infectious diseases that are already endemic in a particular region may spread to form an outbreak. Within the first few weeks after the earthquake, a rapid risk assessment should be conducted by gathering information on the earthquake-affected area and population, with an emphasis on internally displaced people, as well as on the risk of infectious diseases’ emergence and disruptions to public health infrastructure.

## 5. Conclusions

Outbreaks of infectious diseases are often major public health issues following disasters caused by natural hazards. The destruction of local healthcare infrastructure in conjunction with inadequate emergency and preparedness plans can endanger the prompt management and effective treatment of severe health issues and facilitate the occurrence and rapid spread of infectious diseases.

On 6 February 2023, two earthquakes occurred within the southwestern part of the East Anatolian Fault Zone and devastated the southeastern part of Turkey and the northern part of Syria. Shortly after the earthquakes, many risk factors for the emergence of infectious diseases have been concentrated in the affected areas of both countries. Tens of thousands of people were displaced, since their homes collapsed or suffered severe structural damage, making it impossible for them to return. Lack of access to safe drinking water, overcrowding and poor sanitation attributed to destruction of lifeline infrastructures are major risk factors for the emergence of infectious diseases in the earthquake-affected areas of East Anatolia. In addition, the coexistence and synergy of disasters and crises of different origin including war conflicts, the evolving COVID-19 pandemic and the cholera epidemic complicate an already fragile humanitarian situation, with people in northern Syria facing another harsh winter, unstable security conditions and the risk of cross-border aid being cut off.

Understanding the risk factors that underlie the emergence and transmission of infectious diseases can improve the development and implementation of more effective preventive measures. Effective surveillance of diseases at both local and regional levels is a fundamental prerequisite for early warning and protection against emerging infections and potentially uncontrollable disease transmission in the earthquake-affected areas of the East Anatolia. Furthermore, for the prevention and management of water-borne, rodent-borne, respiratory and skin infectious diseases good practices and lessons learned from previous compound disasters should be applied in a frame of a multi-hazard approach. They comprise rapid and timely provision of basic and critical supplies and meeting of temporary housing needs, provision of sufficient quantities of appropriate medical supplies as well as implementation of vaccination programs and hygiene awareness-raising and sanitation promotion methods.

## Data Availability

No new data were created or analyzed in this study. Data sharing is not applicable to this article.
